# An overview of sickle cell disease from the socio-demographic triangle - a Nigerian single-institution retrospective study

**DOI:** 10.11604/pamj.2022.41.161.27117

**Published:** 2022-02-23

**Authors:** Ogbonna Collins Nwabuko, Uwa Onwuchekwa, Okechukwu Iheji

**Affiliations:** 1 Department of Haematology and Blood Transfusion, Federal Medical Centre, Umuahia and Abia State University, Aba Campus, Abia State, Nigeria,; 2 Division of Nephrology, Department of Internal Medicine, Abia State University Teaching Hospital, Aba, Nigeria,; 3 Department of Internal Medicine, Division of Cardiology, Federal Medical Centre, Umuahia, Abia State, Nigeria

**Keywords:** Sickle cell disease, socio-demographic triangle, hemoglobin variants, epidemiologic triad model, Nigeria

## Abstract

**Introduction:**

Sickle cell disease (SCD) is a hereditary red blood cell disorder of public health importance globally with Nigeria the epicenter zone in Africa. There is a paucity of knowledge on how hemoglobin variants, personal characteristics, and environment (socio-demographic triangle) interact to influence SCD propagation. A clinical overview of these epidemiologic parameters may proffer strategies for controlling the SCD disease burden. The objective of this study was to examine the prevalence patterns of SCD, including other associated epidemiologic and hematological (i.e., hemoglobin concentration, ABO blood groups) parameters from laboratory data.

**Methods:**

this was a retrospective cross-sectional study of 138 newly diagnosed SCD patients in the laboratory unit of the department of haematology using routine alkaline cellulose acetate hemoglobin electrophoresis technique from 2013 to 2014. Demographic and other relevant data were obtained from case notes and laboratory records at the presentation. The agent-host-environment variables were used in the construction of the epidemiological triad chain of transmission.

**Results:**

a total of 138 (1.63%) newly diagnosed SCD patients aged 7 months to 41 years made up of 39% (0.63% SCD prevalence) adults and 61% (1% SCD prevalence) pediatric age-groups were seen out of 8457 consecutive patients screened within the study period. About 98.55% and 1.45% were homozygous sickle-hemoglobin (SS) and heterozygous sickle-hemoglobin C (SC) variants, respectively. The pediatric department (CHER+CHOP) recorded the highest proportion of SCD (65%). In contrast, the public health department had the least proportion (1%). There was a statistically significant difference in the gender status and the months of SCD diagnosis (p=0.0147). The month of April had the highest proportion of SCD. A majority (66.7%) of the SCD had moderate grade anemia.

**Conclusion:**

the study revealed a gender disparity in the months of SCD diagnosis. However, there was no statistical difference in the pediatric and adult SCD prevalence patterns.

## Introduction

SCD is an autosomal recessive genetic disorder of red blood cell which is transferable from parent carriers [(AS father) and (AS mother)] to their offspring [1]. It is a very common hemoglobinopathy of public health importance globally [2,3]. SCD is caused by a point mutation of the nucleotide responsible for the synthesis of glutamic acid resulting in its substitution for valine at the sixth amino acid position of the beta-globin [4,5]. In the de-oxygenated state, the sickle red blood cell becomes sticky and loses the physiological properties of an ideal red blood cell, leading to cascade of problems that may give rise to sickle cell crises and complications.

About 50 million people are living with SCD globally and Nigeria is the epicentre zone with about 4-6 million people living with the disease (1 in every 4 Nigerians has a sickle cell trait) [6]. Annually, about 300,000 newly diagnosed SCD children are born worldwide. Sub-Saharan Africa contributes about 75% of the number [7]. Nigeria accounts for 100,000-150,000 newborns living with SCD annually (33% of the global burden of SCD) [8]. Therefore, Nigeria occupies a strategic position in the epidemiology of SCD from the global perspective. The prevalence of SCD within the states in Nigeria ranges from 1%-3% [9,10]. Hb-SS is the predominant hemoglobin variant found in Nigeria while Hb-SC occurs sporadically, especially in the south-western Nigeria [11].

SCD poses significant challenges to the global population health. It contributes significantly to the morbidity and mortality of pediatric and adult population. About 50%-90% of children born with SCD in low- and low-middle-income countries of sub-Saharan Africa die before their fifth birthday [6]. It accounts for 20% of neonatal mortality and 5% of mortality of under-5 children in the african continent [12,13]. It is contributory to several obstetric complications and high maternal mortality rates of women of child-bearing age living with SCD in the region [14,15].

The economic burden of SCD could be quite enormous to the family of sufferers and the nation [16-18]. Although SCD is a genetic disease, socio-demographic characteristics of the host person plays significant role in predicting the direction of the disease trajectory [19]. There is a web of connectivity between the causal risk factors and the socio-demographic characteristics of the host. The epidemiologist, in collaboration with the hematologist, uses the causal inferences to either break the chain of sickle cell gene or control the disease burden. This is achievable using the epidemiological triad model [20].

The epidemiological triad model is one of the frameworks used in public health in decision-making on chain of disease transmission. This model has the ability to identify the causal-relationship of a disease process from the agent-host-environmental perspective and the possible strategic interventions for prevention or controlling it. Two epidemiological triad models used in public health are traditional epidemiological triad model useful in communicable diseases resolution [21] and the Millers modified epidemiological triad model applicable in non-communicable disease (for example SCD) [20].

**Figure 1:**
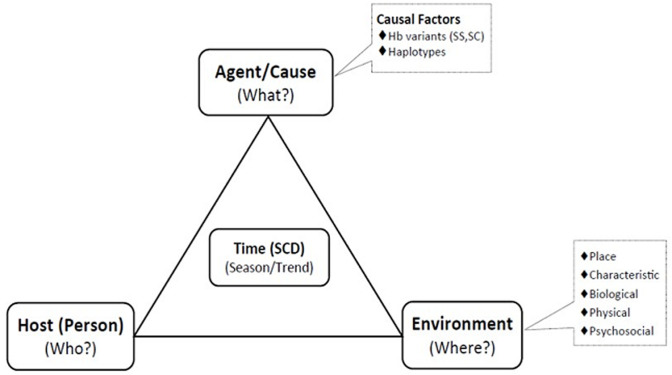
Miller´s modified epidemiologic triad model for descriptive epidemiological analysis of SCD

The Millers modified epidemiological triangle has three vertices namely the agent (what), the person (who), and the environment (where). Time (also known as the “when”) is the intersection point of the three vertices. These vertices tend to demystify some questions about a disease condition (SCD in this case). The “agent factors” (also known as the “what”) in this context connote the various genetic-related risk factors (hemoglobin variables of SCD) with direct or indirect impact on the sickle cell gene that could predispose or aggravate SCD. The “host factors” (otherwise known as “who”) are those personal characteristics vulnerable to SCD or its complication. This is where population demographics (i.e., age, gender, educational status, marital status, occupation) come into play in the epidemiology of SCD. The “environmental factors” (also known as “where”) include the place characteristics, biological, physical and psychosocial environments that may impact SCD clinical course or determination. This is where the social determinants of health play significant role in predicting SCD trajectory. The “time” (also known as “when”) in the modified epidemiological triad model of SCD is the intersection point of the three vertices (what, who and where). The time describes the trending pattern and the seasonality of SCD in a given population (Figure 1) [20].

SCD is a genetic disease controllable by modification of the socio-demographic behaviour of the sufferer. Hence, the socio-demographic characteristic of a person living with SCD is a key player in determining his possible health outcome. It is a topmost priority in budgeting and allocation of resources; restoration of healing and quality of life of people living with SCD. Modification of the socio-demographic dynamics is a strategic leadership approach of minimizing complications of SCD. The modification of socio-demographic behaviour of the target population and the pathogenic basis of the disease are the proposed new approaches of breaking the abnormal sickle cell gene transmission in modern medicine.

This study examines the socio-demographic characteristics of a population of sickle cell disease patients in a tertiary health institution in South-eastern Nigeria using the Millers modified epidemiological triad model. In this study, the hemoglobin variants of the SCD patients represented the agent factors (“what”), the age and gender of the study population represented the host factors (“who”), the referring clinical departments of the patients represented the environmental factors (“where”) while the trending pattern of the SCD at different months of the year represented the time (“when”). This was a descriptive epidemiological overview of the role of socio-demography as a preventive, control and prognostic intervention in SCD trajectory. The study aimed at critically examining the socio-demographic characteristics of SCD patients enrolled at a tertiary health center and how such parameters could be used to improve the health indices of the target population.

## Methods

**Study site:** this study was carried out at the Federal Medical Center, Umuahia (FMCU), Abia State, South-Eastern Nigeria. It is a 350-bed tertiary health center. It is a center of excellence in the research and management of sickle cell disease in the region. The hospital serves as a referral center for inhabitant of Abia State and their neighboring states of Imo, Anambara, Enugu, Ebonyi, Cross Rivers, Akwa-Ibom, and Rivers States. These states account for about 20% of the Nigerian population based on 1991 population census [22,23].

**Study design and setting:** a retrospective cross-sectional study encompassing 8457 consecutive patients who presented at the BGS laboratory unit of the department of haematology, FMCU for genotype/phenotype tests. The period of recruitment was between January 2013 and December 2014. A post-structured questionnaire made up of 4 major sections that met the agent-host-environment criteria for epidemiological triad model was crafted from the demographic and other relevant data obtained from the patients´ laboratory records. The 4 major sections of the questionnaire are: i) the agent factors (i.e., hemoglobin variant); ii) the personal characteristics, also known as host factors (i.e., age, gender); iii) the environment (i.e., referring clinical department of FMCU); iv) the time the test was conducted.

**Participants:** a total of 8457 patients were seen during the study period out of which 138 were SCD. This was a fair representation of the prevalence of SCD in the population size studied. The inclusion criteria for subjects´ selection were: i) the participants must be newly diagnosed SCD irrespective of age; ii) the participant must be a referral from the clinical department of FMCU. The exclusion criteria included participants who are non-SCD or who did not meet the above inclusion criteria for selection. The sample size was generated based on those who met the inclusion criteria for eligibility. The clinical departments of FMCU served as the sources of participants recruited for this study. They were: accident and emergency (A&E), ante-natal clinic (ANC), children emergency room (CHER), children out-patient (CHOP), general out-patient department (GOPD), hematology (HAEM), public health department (PHD) and surgical out-patient department (SOPD).

**Variables:** five major variables were used in study outcome prediction. They include: the causative agent variables which in this study is the hemoglobin variants (i.e., SS and SC); the host variables or biodata (i.e., age, gender); environmental variables (i.e., referral clinical departments such as A/E, CHER, CHOP, GOPD, HAEM, ANC, PHD and SOPD); seasonal (time) variables (January-December) and causative agent-related variables such as anemia grades based on levels of hemoglobin (Hb) concentration such as mild (Hb = 10.0-12.9 g/dL), moderate (Hb = 7.0-9.9 g/dL) and severe (Hb= <7.0 g/dL); ABO-Rhesus blood groups (Groups A, B, AB, O, Rh positive or Rh negative) sickle cell trait (AS) and non-SCD hemoglobin variant (i.e., AA). The dependent variables in this study were referral clinical departments, time of diagnosis of SCD during the study years, while independent variables were age and gender.

**Survey procedure:** a Miller’s modified epidemiological triad model of “What”, “Who”, “Where” and “When” was used to explore the descriptive epidemiology of newly diagnosed SCD patients in FMCU, a south-eastern tertiary health center. The “What” in this context connotes the causal factors of SCD, which in this study was represented by the SCD hemoglobin variables (i.e., SS or SC) of SCD target population. The “Who” in this study explored the personal characteristics of the target population, which in this case was the disease prevalence patterns in the age (pediatric and adult), and gender (Male and Female) categories. The “Where” in the study represented the environmental or external modifying factors that will affect the health indices of the study population, (synonymous to “social variables” or social determinants of health) which in this study was represented by the referring clinical departments of the patients. The “When” connotes the seasonal (time) pattern of SCD in both the pediatric and adult SCD population. This study used the epidemiological triad model to graphically describe the clinical epidemiology of SCD (Figure 1).

**Data analysis:** data was coded and entered into the excel sheet. Data analysis was using SAS JMP Statistical Discovery Software version 14.3 (SAS Institute, Cary, North Carolina, USA). Descriptive statistics such as mean, standard deviation, frequency, graphs were used to summarize all continuous and categorical variables data. The chi square test was used to establish the relationship between the dependent and independent variables. The following groups were compared: age status by gender, age status by referral clinical departments, age status and gender by months of the year of diagnosis. Statistical significance was set at probability (P) = 0.05.

**Ethical considerations:** this was obtained from the institutional review board of FMCU, in keeping with the guidelines of the declaration of Helsinki (1964) and Good Clinical Practice. The Health Research Ethics Committee (HREC) identification number is FMC/QEH/G.596/Vol.10/453. No verbal or written informed consent was obtained from the patients as this was a retrospective study. However, the patients´ identities were kept confidential.

## Results

### Descriptive statistics of socio-demographic and hemoglobin-related variables

Eight thousand four hundred and fifty-seven patients were seen during the study period. The participants were made up of more females (77%, n=6512) than males (23%, n=1945) with a M: F ratio of 1: 3. The study included both children and adults whose ages ranged between 7 months - 49 years. Three hemoglobin variants were identified as follows: AA (76.19%, n=6444), AS (22.18%, n=1876), SS (1.61%, n=136) and SC (0.02%, n=2). Among the 138 SCD hemoglobin variants, SS was more predominant (98.55%, n=136) than SC (1.45%, n=2) (Table 1). The ages ranged between 7 months - 41 years with overall mean age of 14.8±11.8 years (mean pediatric SCD age 6.8±4.2 years; mean adult SCD age 27.8 ±5.5 years). Majority of SCD sub-population were females (56.6%, n=64) compared to male (43.4%, n=49) with M: F of 1: 1.3. More pediatric SCD (61%, n=69) was recorded compared to adult SCD (39%, n=44) with Adult-Pediatric ratio of 1: 1.5 (Table 2). Translating this into prevalence, it means a pediatric SCD prevalence of 1.0% and adult SCD prevalence of 0.63%. The pediatric department (made up of CHER and CHOP) had the highest proportion of SCD patients (65%, n=65) while the public health department (PHD) had the least proportion (1%, n=1) (Table 3). A few hemoglobin concentrations (13%, n=18) were recorded among the participants during the study period. Majority of the studied participants had moderate grade anemia (66.7%, n=12), followed by severe grade anemia (27.8%, n=5). The least grade of anemia was the mild grade anemia (5.5%, n=1) (Table 4). Moderate- and severe-grades of anemia were relatively higher in the adult age group at (55.6%, n=5) and (60%, n=3) respectively. The male gender (63.6%, n=4) had more moderate grade anemia, while the female gender recorded more severe grade (4(80%). The various clinical departments with severe grades of anemia were A/E, ANC, CHER, GOPD and HAEM departments (Table 5). Few (23.2%, n=32) ABO blood groups were identified among the study participants and their patterns of spread were blood groups O (56.3%, n=18), A (34.4%, n=11) and B (9.4%, n=3). All identified ABO blood groups were rhesus-D (Table 5).

**Table 1:** pattern of hemoglobin variants of blood samples collected from patients seen during study period

Serial Number	Hb variant (n=8457)	Frequency (n=8457)	Percent (%)
1.	AA	6447	76.19
2.	AS	1876	22.18
3.	SS	136	1.61
4	SC	2	0.02

Hb = Hemoglobin

**Table 2:** socio-demographic characteristics of SCD in the study population

Socio-demographic characteristics	Pediatric Number n (%)	Adult Number n (%)	Total
**Age (years) Mean ±SD**	6.8±4.2	27±5.5	14.8±11.8*
**Gender**			
Male	31 (49)	18 (36.7)	49
Female	38 (59)	26 (40.6)	64
**Total**	69	44	113

Chi-square, x2, df, (p-value) 0.177, 1, p=0.6740; *Overall Mean Age; SD: Standard deviation

**Table 3:** distribution pattern of SCD based on referral clinical departments

Characteristic	Frequency (n=100)	Percentage (%)
Referral Clinical Dept.(n=100)^a^		
A/E	7	7.00
ANC	7	7.00
CHER	48	48.00
CHOP	17	17.00
GOPD	15	15.00
HAEM	2	2.00
PHD	1	1.00
SOPD	3	3.00

a Missing data A/E = Accident and emergency; ANC = Antenatal clinic; CHER = Children emergency; CHOP = Children out-patient; Dept. = Department; GOPD = General out-patient; HAEM = Haematology; PHD = Public Health; SOPD = Surgical out-patient

**Table 4:** anaemia grades in the study sub-population

Anaemia^a^	Frequency (n=18)	Percentage (%)
Mild	1	5.5
Moderate	12	66.7
Severe	5	27.8

a Missing data

**Table 5:** distribution of anaemia and socio-demographic parameters of study population

Anaemia grades (n=18)	AGE (n=14)	SEX (n=17)	Blood group (n=6)		Clinical department (n=15)										
			PAED	AD	M	F	A	B	O	A/E	ANC	CHER	GOPD	SOP	HAEM
Mild			0	0	0	1	0	0	0	0	0	0	0	0	0
Moderate			4	5	4	7	2	0	3	0	3	4	1	2	0
Severe			2	3	4	1	0	1	0	1	1	1	1	0	1
Total			6	8	8	9	2	1	3	1	4	5	2	2	1

AD: Adult; A/E: Accident and emergency; ANC: Antenatal clinic; CHER: Children emergency; CHOP: Children out-patient; F: Female; GOPD: General out-patient; HAEM: Haematology; M: Male; PAED: Pediatric; PHD: Public Health; SOPD: Surgical out-patient

### Comparative analysis of host variables with the environmental and seasonal variables

**Figure 2:**
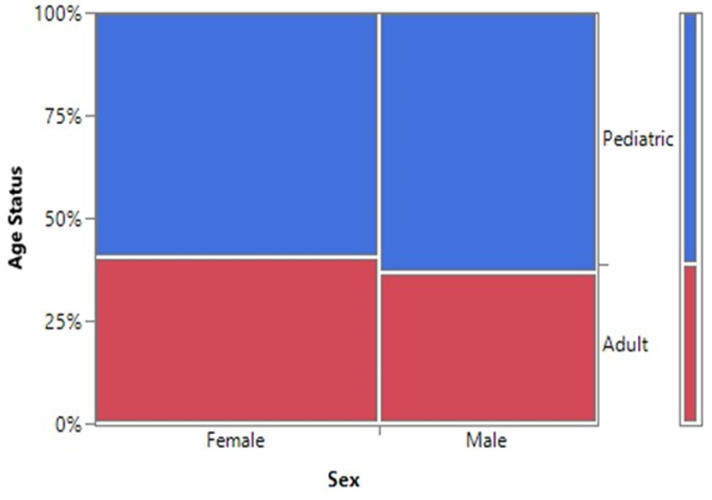
a mosaic plot showing the proportion of age status and gender of SCD patients seen at FMCU

**Figure 3:**
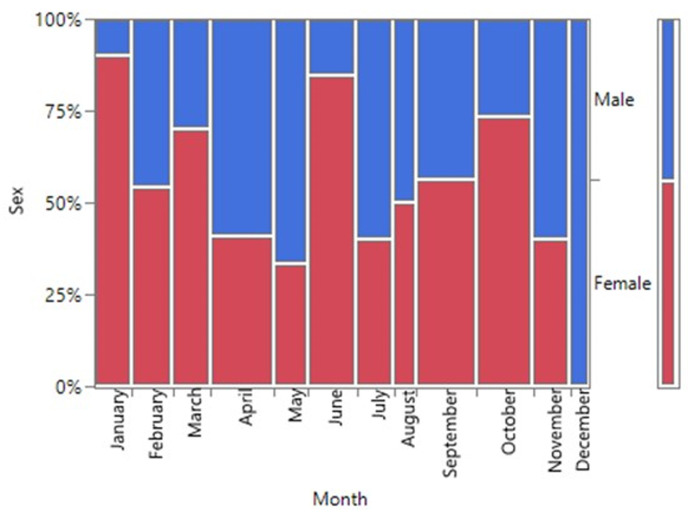
a mosaic plot showing gender distributions by months of patients diagnosed SCD at FMCU between January to December

**Figure 4:**
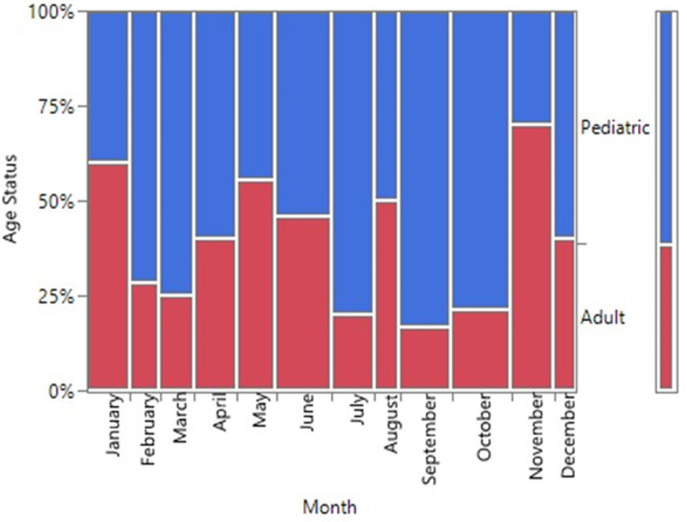
a mosaic plot showing the proportion of SCD by months (January-December) and age status

There was no statistical difference between the age status (Pediatric and Adult) and the gender of SCD patients seen at FMCU. (p=0.6740) (Figure 2, Table 2). There was a statistically significant difference in the gender status and the months of diagnosis of SCD from the study (p=0.0147). The month of April had the highest number of SCD (12.9%, n=17) while the least was December (5%, n=6). Male prevalence was statistically significantly higher in the month of April, (7.6%, n=10) while that of female gender was bimodal in June (8.3%, n=11) and October (8.3%, n=11) (Figure 3). Although the highest proportion of pediatric and adult newly diagnosed SCD were recorded in the months of October (9.7%, n=11) and November (6.1%, n=7) respectively, there was no significant difference in the proportion of SCD by month and age status (p=0.2118) (Figure 4).

**Missing data**: about thirty-seven, 38 and 25 participants had missing data for age status, referral clinic, and gender respectively. The age status of one and 2 mild- and moderate grades anemic SCD participants were respectively missing. Seven, 4 and 1 ABO-Rhesus blood groups of moderate-, severe- and mild-grade anemic SCD participants were respectively unascertained. The referral clinical departments of one mild-grade anemic SCD and 2 moderate-grade counterparts were missing. These were due to the retrospective nature of this study.

## Discussion

The socio-demographic profile of people living with SCD is a strong predictor of the clinical course and outcome of the disease. This is very important considering Africa as one of the continents with the least Human Development Indices (HDI) [16,17,19]. The modified epidemiological triad model was the most suitable approach to address this issue by breaking or modifying the agent-host-environment chain of transmission. A SCD prevalence of 1.63% was in keeping with the national prevalence range of 1%-3% in Nigeria [10,24,25]. However, it was higher than the value reported by Nnaji *et al*. (0.98%) among prospective premarital couples in Southeastern Nigeria [26], but lower than values recorded in the northern Nigeria [24]. These relative differences in SCD prevalence between the southern and northern Nigeria could be attributable to the disparities in awareness, knowledge, perceptions and cultural practices with respect to pre-genetic counseling in the multi-ethnic groups that make up Nigeria [24,26]. The two hemoglobin variants SS and SC were the commonest SCD hemoglobin variants found in Nigeria. A 98.6% prevalence of homozygous SS was in keeping with previous studies which showed predominance of hemoglobin SS variant in Nigeria [10,24,27]. Previous studies showed that while hemoglobin SS is more evenly distributed in all the geopolitical zones of Nigeria, hemoglobin SC is less distributed; more prevalent in western Nigeria, where it could accounts for about 3-4% of hemoglobin variant of SCD, but least in the east of the river of Niger delta [25,28]. The severity of SCD is worse with homozygous SS (SCA) compared to heterozygous SC due to the mild grade, lesser crises and haplotype associated with sickle-hemoglobin C disease [25].

The overall mean age of 14 years clearly depicts that SCD is still predominated by pediatric age status. The pediatric and adult mean ages of 6 years and 27 years were in keeping with previous studies across the geo-political regions of Nigeria and Ghana [7,10,24,25,29,30]. The relative equal gender ratio from the study was similar to that found in previous similar studies [24,29,30]. The study showed that the proportion of SCD among the pediatric age group was not statistically significant higher than the adult counterpart. This may be an indication of reduction in mortality rate of SCD as the disease advances from childhood to adulthood. This was in contrast to previous studies which showed that 50%-80% of children born with SCD in low-middle-income countries of sub-Saharan Africa die before their 5th birthday [6,12,13]. This could be explained by the gradual improvement in care of people living with SCD in the region. Increasing the adult-pediatric ratio of SCD is a strategic approach of breaking the chain of its transmission.

With respect to the referral clinical departments, the pediatric department had the highest prevalence of SCD while the public health department had the least prevalence. A 1% prevalence from public health department shows that the level of awareness of SCD in the environment is still very low. Most referrals for hemoglobin phenotype (genotype) tests are incidental (i.e., secondary to illness, accidental, reactionary) and not as a primary protective (screening) intervention, which ideally should emanate from the public health department, a clinical department saddled with the responsibility of health awareness creation and screening tests.

Most SCD patients from pediatric department come through the children emergency unit (CHER) confirming that their diagnoses were accidental. There was no newborn screening (i.e., diagnosis of SCD made within 1^st^ month of life) recorded in this study. The earliest diagnosis was at the 7^th^ month of age. Early diagnosis of SCD is still a far cry in Nigeria. This could be the reason for increase in complications and mortality rates of people living with SCD in the region [14,15,31]. While the mortality rate of SCD in high-income countries has reduced to less than 1% due to improved case ascertainment (early diagnosis) and therapeutic interventions, the reverse is the case in low- and low-middle-income countries of sub-Saharan Africa, where SCD is the 6th leading cause of death and accounts for about 5%-16% of under 5 mortality [12,13,28,31]. While the life expectancy of people living with SCD ranges between 53-60 years in developed countries, it is still less than 50 years in a developing country such as Nigeria [6,28].

The gender distribution of SCD by months of the year was statistically significant. There are five important months with respect to seasonality (time factor) of SCD from this study, namely April, June, October, November and December. The simplest explanations of the trending patterns of SCD in the study could be the events that characterize the peak periods. The earliest marriage season of every year comes in April for the Christian religion, while the late season preparation starts from October. This could be possible explanations for the high proportion as more prospective couples go for hemoglobin phenotype tests to determine their compatibility [26]. Similarly, the preparation for new school year comes with hemoglobin phenotype tests for the prospective students. These procedures occur predominantly in June and October and could probably account for the high proportion of SCD in the pediatric age groups. Anemia is the commonest complication encountered by people living with SCD in Nigeria. Moderate grade anemia is the most common grade of anemia from this study. This was in keeping with retrospective studies on pregnancy in SCD and non-SCD conducted in the Niger delta Nigeria [32,33]. Severe grade anemia in SCD was commoner in the female gender while moderate grade was commoner with the male gender from the study. However, these findings need a more robust population size in order to be proven scientifically.

**Limitations:** the study was limited by lack of facilities to determine the haplotypes of sickle cell anemia as this would have contributed in the evidence-based findings especially in demystifying the causal factors of severity of various hemoglobin variants in SCD. Additional data on the host factors such as educational level, marital status, socioeconomic status, and occupation/social class of the study population could have given more insight into the socio-demographic characteristics of the study population. There is likelihood that some hemoglobin variants were missed using the old hemoglobin electrophoresis technique. A more verifiable result could have been obtained if the modern technique such as high performance liquid chromatographic (HPLC) equipment was used in identification of hemoglobin variants. The retrospective nature of the study design made it impossible to obtain enough data that could have been useful in decision-making. The post-structured questionnaire used in the study design is less preferable to pre-structured questionnaire used in high evidence-based observational studies. These limitations whereby some referral clinical departments without corresponding ages were accounted for could directly or indirectly contribute to bias outcome predictions, hence compromising the generalizability of study findings.

## Conclusion

The study revealed a significant gender disparity of SCD diagnosed during the months of the year. However, there was no significant disparity in pediatric (1%) and adult (0.63%) SCD prevalence patterns in the region. Late diagnosis of SCD is a major public health challenge in Nigeria. The determinants of SCD in Nigeria are predominantly incidental (secondary) as against the primary health protective approach such as newborn screening tests obtainable in high-income countries. Health illiteracy, poor legislation, late detection and predominance of homozygous sickle-hemoglobin variant are predictive markers of poor prognostic outcome of SCD. Early detection of SCD is paramount to reduce its measles, mumps, and rubella (MMR) and improve the *quality-adjusted life-year* (QALY). Therefore, health promotion of need to enact policies that will promote education, newborn screening, researches and other therapeutic interventions becomes necessary. This is the strategic approach to improve health indices of SCD sufferers in Nigeria.

### What is known about this topic

- Sickle cell is a hereditary red blood cell disorder;
- Socio-demographic characteristics have influence on health outcome of SCD.

### What this study adds

- There is a statistically significant gender disparity of SCD diagnosed during the months of the year in FMCU;
- There is no statistical significant difference in the pediatric and adult SCD prevalence patterns;
- Majority of the SCD population seen during the study period had moderate grade anemia.
